# Multicomponent Solar Cells with High Fill Factors and Efficiencies Based on Non-Fullerene Acceptor Isomers

**DOI:** 10.3390/molecules27185802

**Published:** 2022-09-07

**Authors:** Qiuning Wang, Yiwen Hou, Shasha Shi, Tao Yang, Ciyuan Huang, Shangfei Yao, Ziyang Zhang, Chenfu Zhao, Yudie Liu, Hui Huang, Lihong Wang, Chaoyue Zhao, Minghui Hao, Ye Tian, Bingsuo Zou, Guangye Zhang

**Affiliations:** 1 College of New Materials and New Energies, Shenzhen Technology University, Shenzhen 518118, China; 2 Julong College, Shenzhen Technology University, Shenzhen 518118, China; 3 Guangxi Key Laboratory of Processing for Nonferrous Metals and Featured Materials, Ministry of Education, School of Resources, Environments and Materials, Guangxi University, Nanning 530004, China; 4 Key Laboratory of New Processing Technology for Nonferrous Metals and Materials, Ministry of Education, School of Resources, Environments and Materials, Guangxi University, Nanning 530004, China; 5 Centre for Mechanical Technology and Automation, Department of Mechanical Engineering, University of Aveiro, 3810-193 Aveiro, Portugal; 6 Suzhou Key Laboratory of Advanced Lighting and Display Technologies, School of Electronic and Information Engineering, Changshu Institute of Technology, Changshu 215500, China

**Keywords:** multicomponent organic solar cells, power conversion efficiency, non-fullerene acceptor isomers, fill factor, generality

## Abstract

Multicomponent organic solar cells (OSCs), such as the ternary and quaternary OSCs, not only inherit the simplicity of binary OSCs but further promote light harvesting and power conversion efficiency (PCE). Here, we propose a new type of multicomponent solar cells with non-fullerene acceptor isomers. Specifically, we fabricate OSCs with the polymer donor J71 and a mixture of isomers, ITCF, as the acceptors. In comparison, the ternary OSC devices with J71 and two structurally similar (not isomeric) NFAs (IT-DM and IT-4F) are made as control. The morphology experiments reveal that the isomers-containing blend film demonstrates increased crystallinity, more ideal domain size, and a more favorable packing orientation compared with the IT-DM/IT-4F ternary blend. The favorable orientation is correlated with the balanced charge transport, increased exciton dissociation and decreased bimolecular recombination in the ITCF-isomer-based blend film, which contributes to the high fill factor (FF), and thus the high PCE. Additionally, to evaluate the generality of this method, we examine other acceptor isomers including IT-M, IXIC-2Cl and SY1, which show same trend as the ITCF isomers. These results demonstrate that using isomeric blends as the acceptor can be a promising approach to promote the performance of multicomponent non-fullerene OSCs.

## 1. Introduction

Organic solar cells (OSCs) have drawn wide interest for the past decade thanks to the potential semi-transparency, flexibility, and solution processability [1,2,3,4,5]. Currently, the power conversion efficiencies (PCEs) of binary OSCs, whose active layers comprise one electron donor and one electron acceptor, have surpassed 18% via materials design and device engineering [6,7,8,9,10,11,12,13,14,15]. However, organic semiconductors used in solar cells typically have a thickness of only a few hundred nanometers with a relatively narrow absorption range in the visible spectrum owing to the molecular excitonic nature of the primary excitation [16]. The narrow absorption of organic semiconductors limits the PCE of the binary solar cells. To solve the above issue, the idea of multicomponent OSCs, which involves more than two materials fabricated in the active layer, has recently been proposed and practiced by many studies [17,18,19,20,21,22]. A combination of three (ternary) or even four (quaternary) typical organic semiconductors in the solar cells gives multicomponent OSCs various beneficial features such as broadened absorption profile, simplicity of single-junction OSCs. Therefore, multicomponent OSCs are considered a simple and effective way to enhance light harvesting and conversion. With the emergence of highly efficient non-fullerene acceptors (NFAs) [23,24,25,26,27,28,29], one polymer donor and two NFAs have become the prevailing method for the active layer of ternary OSCs [30,31,32]. Consequently, PCEs over 18% have been achieved in ternary solar cells [33,34,35,36,37].

A typical NFA consists of a planar donor-core containing outstretched side chains and electron-withdrawing end-capping groups (EGs) [38,39,40,41,42,43,44,45,46], e.g., the widely-used small molecule ITIC. In some cases, the EGs are a mixture of two isomers due to synthetic reasons [47,48,49,50,51]. As a result, the NFA contains three isomers that are hard to separate from each other in principle. Recently, our group has demonstrated that the use of two structurally similar NFAs provides the ternary blend film with favorable domain size and relatively high domain purity, thereby delivering an improved fill factor (FF) and therefore a high device performance. In this regard, isomers natively have the advantage of high structural homogeneity, which would be an intriguing strategy to fabricate multicomponent blend towards high-performance solar cells. However, this strategy has never been explored until now. 

**Scheme 1:**
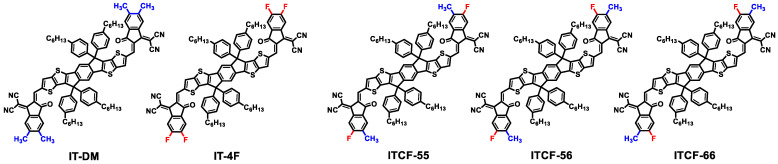
The chemical structures of the small molecules acceptors used in this work.

In this work, we fabricated a multicomponent blend solar cell based on our recently developed NFA, i.e., ITCF [50] as the acceptors and the polymer, J71, (Scheme S1, Supplementary Materials) as the donor. In principle, ITCF consists of three isomers, ITCF-55, ITCF-66, and ITCF-56 (Scheme 1) according to the position of the methyl unit and the fluorine atom, which cannot be separated due to the similar polarity. Compared to IT-DM versus IT-4F, the difference among ITCF isomers comes only from the variation in the position of the substituents (methyl and fluorine atom) on the end group. Therefore, the structural homogeneity among ITCF isomers is higher than that between IT-DM and IT-4F. In this paper, the ternary solar cell based on the donor polymer J71 and the acceptor blend of IT-DM and IT-4F (Scheme 1) was fabricated to obtain a comprehensive understanding of the solar cells based on isomers. Note that the weight ratio of IT-DM/IT-4F was set to 1:1 because only in this way can we have the same element content as ITCF. In addition, J71:IT-DM- and J71:IT-4F-based binary solar cells were also fabricated. When the two structurally similar SMAs (IT-DM and IT-4F) were blended with the donor polymer J71, the open-circuit voltage (*V*_OC_) and the short-circuit current density (*J*_SC_) of the corresponding ternary solar cell are between those of the two binary cells. The high FF from the IT-4F binary cell provides the ternary cell with a slightly higher PCE than that of binary cells. We found out that ITCF-isomer-based OSCs have better charge transport, suppressed recombination, and an improved morphology with higher crystallinity than those of IT-DM/IT-4F-based ternary solar cells. These beneficial characteristics enabled a high FF of 77.3% and a PCE of 13.4%. Both the FF and PCE of the ITCF device outperform the non-isomer-based ternary cell. To assess the generality of this approach, we fabricated other ternary devices using acceptor isomers including IT-M isomers, SY1 isomers and IXIC-2Cl isomers, which show same trend as the ITCF-isomer-based OSCs. Our results indicate that using NFA isomers that possess high structural homogeneity to fabricate multicomponent solar cells is an efficient approach to achieve a favorable morphology and thus attain an improved device performance, which could be expanded to various different high-performance NFAs.

## 2. Results and Discussion

**Figure 1:**
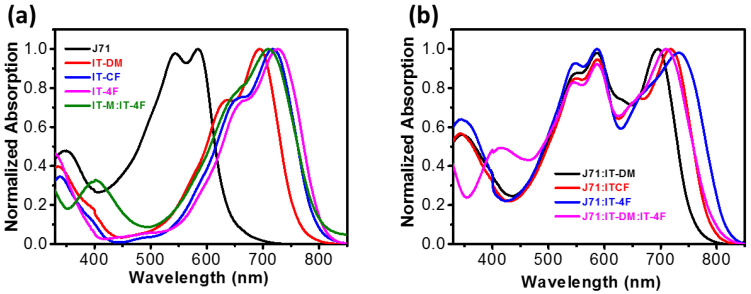
Normalized UV-vis absorption spectra of the films. (**a**) J71 film, acceptor films, and the IT-DM/IT-4F blend film; (**b**) Absorption spectra of the active blend Films. The data for pure IT-DM, IT-CF, IT-4F and for the binary blend of J71:IT-DM and J71:ITCF are used with permission from ref. [50]. Copyright (2019) American Chemical Society.

The absorption spectra of the donor, J71, and the acceptors, as well as the blend film of IT-DM/IT-4F, are presented in Figure 1. The absorption spectra of the pure acceptor films and the blend film of IT-4F/ID-TM compensate for J71. The absorption onset of the IT-4F film is located at 803 nm, which is redshifted by 44 nm compared with that of IT-DM (759 nm), indicating the appropriateness of IT-4F as the guest to the J71:IT-DM system. The optical properties, such as the maxima and the onset of the absorption, of the IT-DM/IT-4F blend film (weight ratio 1:1) are between those of IT-DM and IT-4F, which are similar to those of the ITCF isomers. Once the acceptors are mixed with donor polymer J71, a broadened absorption spectrum covering the entire visible region is realized. The IT-DM/IT-4F blend shows an absorption similar to that of ITCF in the range of 500–800 nm, indicating that it is possible for the two active layers to realize similar *J*_SC_ in solar cell devices. We note that some of the absorption spectra for the pure films and binary blend films are used with permission from our previous report [50]. They are used here just to make the comparison more visible for the ease of readers.

The morphology of the pure films based on the donor polymer, acceptor molecules and the IT-DM/IT-4F are detected by tapping-mode atomic force microscopy (AFM) (Figure S1). The IT-DM film exhibits a rougher surface where root-mean-square (RMS) is 1.43 nm. The IT-4F based film is smoother with the RMS of 0.409 nm. The blend film of them has an RMS of 1.16 nm. Regarding the ITCF isomers film, a relatively smooth surface morphology was found with 0.731 nm RMS. In addition, the J71 film exhibited a similar top surface to that of ITCF with an RMS roughness of 0.615 nm. The crystallinity of the IT-DM/IT-4F blend film was improved compared to the pristine ones, showing strong aggregation and amorphous morphology, respectively. However, the IT-DM/IT-4F blend possesses a rougher surface morphology than the ITCF film, indicating different crystalline properties.

**Figure 2:**
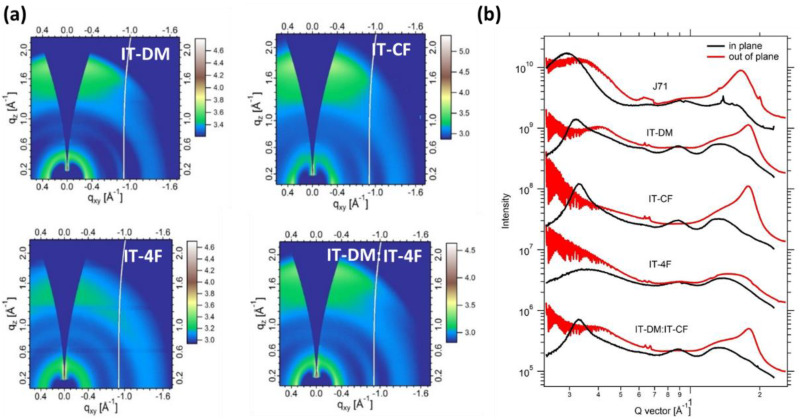
(**a**) The GIWAXS 2D patterns of the pristine films of IT-DM, IT-4F, IT-DM/IT-4F blend and ITCF; (**b**) In-plane (IP) and Out-of-plane (OOP) GIWAXS Line-cut Profiles.

The bulk morphology of J71 and the acceptors films were studied by grazing incidence wide-angle X-ray scattering (GIWAXS) (Figure S2) [52]. The corresponding line-cut profiles are shown in Figure 2. The parameters are summarized in Table 1. J71 shows a strong π-π stacking (010) peak at 1.645 Å^−1^ in the OOP direction, indicating a face-on orientation for it. The IT-DM film shows a strong (010) scattering peak in the OOP direction with the appearance of a (100) peak in the in-plane (IP) direction, suggesting a predominant face-on orientation for IT-DM. The IT-4F shows an amorphous morphology, which is in line with previous literature [53]. The IT-DM/IT-4F blend film shows an IP (100) peak at 0.331 Å^−1^ and a (010) scattering peak in the OOP direction at 1.753 Å^−1^, which are close to those of the IT-DM film (0.322 Å^−1^ and 1.777 Å^−1^, respectively, Table 1), implying that they originated from the IT-DM component. It is noteworthy that the IT-DM/IT-4F blend film showed an increased crystal coherence length (CCL) (15.41 nm, derived from the Scherrer equation) compared with the IT-DM film (10.05 nm), which suggests that mixing the two molecules at the weight ratio of 1:1 increases the crystallinity in this direction. In addition, the π-π stacking distance of the IT-DM/IT-4F blend film (3.536 Å) decreased compared to that of the IT-DM film (3.584 Å), which could promote charge transport. The ITCF film exhibited similar lamellar stacking distance (18.93 Å) and π-π stacking distance (3.554 Å) to those of the IT-DM/IT-4F blend film (18.988 Å and 3.536 Å, respectively). 

To correlate the morphology with electrical properties, mobilities of the films were estimated using the space-charge-limited current (SCLC) method (Figure S3, Table S1) [54]. The IT-DM/ITCF blend film shows a slightly higher electron mobility than the IT-DM film, which is related to its relatively stronger crystalline tendency. ITCF shows an electron mobility (7.46 × 10^−4^ cm^2^ V^−1^ s^−1^) comparable to that of the IT-DM/IT-4F film (7.29 × 10^−4^ cm^2^ V^−1^ s^−1^).

The photoluminescence (PL) spectra of the pure and mixed films were used to detect the quenching. Figure S4 compared the pure-acceptor(s) and blend films, which shows clear PL quenching, implying efficient exciton dissociation.

**Figure 3:**
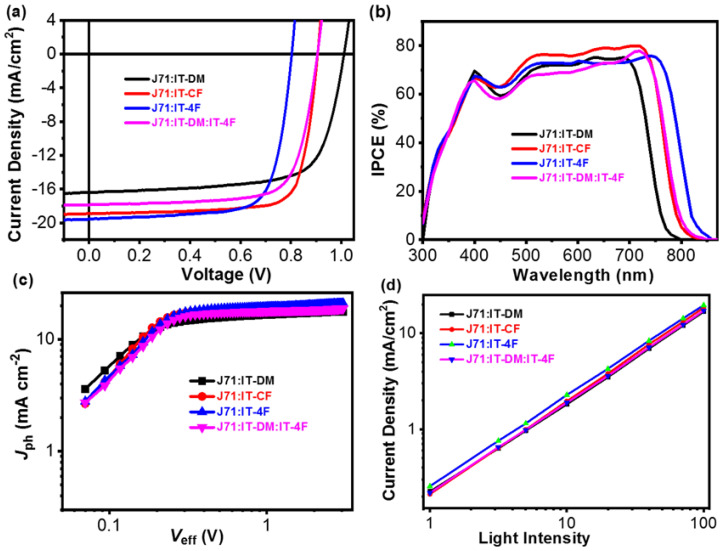
(**a**) *J-V* Curves of the Optimized Devices; (**b**) IPCE Spectra of the Optimized Devices; (**c**) *J*_ph_ vs. *V*_eff_ Curves of the Optimized Devices; and (**d**) *J*_SC_ vs. Light Intensity of the Optimized Devices.

We then made devices in the conventional device architecture: ITO/PEDOT:PSS /J71:ITCF/ZrAcac/Al. The weight ratio of donor/acceptor for the binary solar cell is 1:1. The active layers were prepared by spin-coating the blend solutions (a total blend concentration of 16 mg mL^−1^ in chloroform) at 2000 rpm. The devices were optimized after thermal annealing (TA) at 100 °C for 5 min. The optimized current density-voltage (*J-V*) curves of the OSCs are shown in Figure 3a, and the corresponding photovoltaic parameters are summarized in Table S2. The IT-DM device exhibits a moderate *J*_SC_ of 16.378 mA cm^−2^, an FF of 70.9%, and a high *V*_OC_ of 1.011 V, resulting in a decent PCE of 11.736%, whereas the IT-4F device exhibits a high *J*_SC_ of 19.544 mA cm^−2^, an FF of 73.9%, and a *V*_OC_ of 0.803 V, resulting in a moderate PCE of 11.603%. The higher *J*_SC_ and lower *V*_OC_ of the IT-DM device than the IT-4F device are reasonable, considering the optical bandgap and energy levels of the corresponding acceptors. The ternary solar cell based on IT-DM/IT-4F and multicomponent solar cell based on ITCF isomers were fabricated with the same device architecture as the binary solar cells under the same conditions. The weight ratio of donor and total acceptor molecules in the ternary blend is 1:1. Note that the weight ratio of the two acceptors, i.e., IT-DM and IT-4F, was set to 1:1. Thus, the weight ratio of J71:IT-DM:IT-4F in the active layer is 2:1:1. The *V*_OC_ and *J*_SC_ of the J71/IT-DM/IT-4F ternary device are between those of the IT-DM and the IT-4F binary cells. Due to the FF being as high as that of the IT-4F device, the ternary solar cell delivered a PCE of 11.9%, slightly higher than those of the IT-DM and the IT-4F device. In terms of the ITCF-isomer based device, a high PCE of 13.35% was realized due to the high *J*_SC_ of 18.915 mA cm^−2^ and an outstanding FF of 77.3%. The highest *V*_OC_ (0.905 V) achieved by the IT-DM/IT-4F ternary device is comparable to that of the ITCF device. However, the high *J*_SC_ and FF of the isomer-based device are clearly higher than those of the control devices, resulting in a significantly higher PCE. The *J*_SC_ change is consistent with the incident photon-to-current conversion efficiency (IPCE) spectra. As shown in Figure 3b, the ITCF device demonstrated higher quantum efficiencies than the IT-DM/IT-4F ternary cell in the range of 400–750 nm. The improved photovoltaic performance of the ITCF isomers device relative to the IT-DM/IT-4F ternary device suggests that increasing the structural homogeneity of the NFAs used in the ternary solar cells could impose significant effect on the performance of the devices. 

Photocurrent density (*J*_ph_) versus the effective voltage (*V*_eff_) of the IT-DM/IT-4F ternary and the ITCF isomers OSCs were measured to investigate the overall efficiency of exciton dissociation and charge collection (Figure 3c). The *J*_ph_ is defined as *J*_L_ − *J*_D_, where *J*_L_ and *J*_D_ are the current density under illumination and in the dark, respectively. The *V*_eff_ is defined as *V*_0_ − *V*_a_, where *V*_0_ is the voltage at which *J*_ph_ = 0, and *V*_a_ is the applied bias. The exciton dissociation and charge collection efficiency can be assessed by the ratios of *J*_ph_/*J*_ph,sat_. A summary of these parameters is presented in Table S3. The IT-DM/IT-4F ternary device showed a slightly higher exciton dissociation and a comparable charge collection efficiency (94.7% and 81.7%, respectively) to the parent IT-4F binary device (93.9% and 81.8%, respectively), which is consistent with the two devices showing similar FFs (73.7 for the IT-DM/IT-4F ternary cell and 73.9 for the IT-4F binary cell). The ITCF isomer-based device demonstrated both higher exciton dissociation and higher charge collection probabilities than the IT-DM/IT-4F ternary cell, consistent with their FFs (0.773 vs. 0.737). The higher exciton dissociation and charge collection probabilities of the ITCF isomer-based OSCs could also contribute to its higher *J*_SC_ considering that two cells have similar absorption spectra.

Furthermore, we measured *J*_SC_ vs. light intensity (P_light_) relationship to check the recombination (Figure 3d) [55,56]. The fitted α value is approximately 0.949 for the IT-DM/IT-4F ternary cell and 0.973 for the ITCF isomers cell. The α value of the ITCF isomers device is higher than that of the IT-DM/IT-4F cell, indicative of less bimolecular recombination, which could also contribute to its higher FF.

The charge transport (mobilities) are investigated by the SCLC method (Figure S5). With the results summarized in Table S1, the IT-DM/IT-4F ternary device exhibited a hole mobility (8.68 × 10^−4^ cm^2^ V^−1^ s^−1^) comparable to that of the IT-DM and the IT-4F binary device (8.00 × 10^−4^ cm^2^ V^−1^ s^−1^ and 8.81 × 10^−4^ cm^2^ V^−1^ s^−1^, respectively), suggesting that the J71 molecular arrangement was not seriously disturbed by the third component. The IT-DM/IT-4F ternary device and the IT-4F binary device showed more balanced charge transport than the IT-DM binary device, which could be one of the reasons for their higher FFs. The hole and electron mobilities of the ITCF isomer-based device are similar to those of the other devices, whereas its *μ*_h_/*μ*_e_ (1.57) is the lowest among all devices. The most balanced charge transport is also responsible for its highest FF of 77.3%.

It is known that the *J*_SC_ and FF are closely related to the transport properties of the active layer, which is influenced by the active layer’s morphology [57,58]. First, we studied the surface morphologies of the blend films through AFM (Figure S1). The IT-DM/IT-4F ternary blend film exhibits a smooth surface with an RMS surface roughness (0.904 nm) between those of the IT-DM and the IT-4F binary films (1.62 nm and 0.787 nm, respectively) [50]. The ITCF isomers blend shows a relatively rougher surface (0.956 nm) compared with the IT-DM/IT-4F ternary blends. Furthermore, the phase images show that the IT-DM/IT-4F ternary and the ITCF isomers blend films display well-distributed texture, with the ITCF film having a clear granular texture.

**Figure 4:**
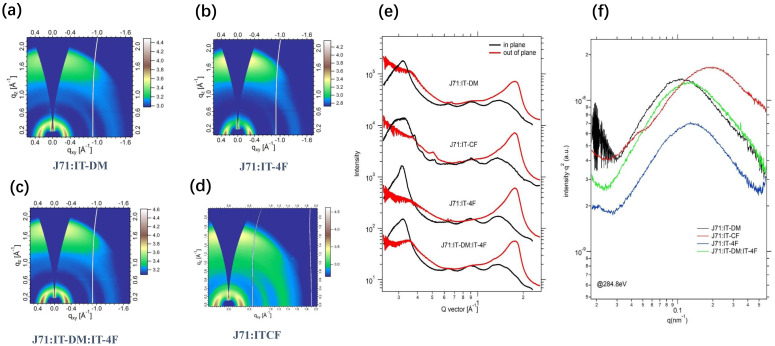
The GIWAXS 2D Patterns of the Active Blend Films of J71:IT-DM (**a**), J71:IT-4F (**b**), J71:IT-DM/IT-4F (**c**), and J71:ITCF (**d**,**e**) In-plane (IP) and Out-of-plane (OOP) GIWAXS Line-cut Profiles of Active Blend Films; (**f**) Soft X-ray Scattering of Active Blend Films.

The molecular packing and orientation of the blend films were investigated by GIWAXS (Figure 4a–d). The corresponding line-cut profiles are shown in Figure 4e. All the blend films exhibited strong lamellar packing (100) peaks in the IP direction and strong (010) π-π stacking peaks, indicative of the predominant face-on orientation relative to the substrate. In order to compare the π-π stacking characteristics of two active blend film in the vertical direction, the (010) CCL was calculated by the Scherrer equation. Since the (010) peaks of the donor polymer and acceptor molecules partially overlap, the contribution from each component to the blend is hard to distinguish. The IT-DM/IT-4F ternary blend shows a CCL value of 1.745 nm, which is a little lower than that of the ITCF isomers blend (2.164 nm). The greater CCL in the OPP direction indicates a higher degree of molecular order formed in the ITCF isomers blend film, which may be caused by the device parameters.

To further understand the microscopic morphology, we carried out resonant soft X-ray scattering (R-SoXS) experiments to probe the phase separation of the active blend films (Figure 4f) [59]. The IT-DM binary blend film shows a scattering peak at *q* = 0.11 nm^−1^, which corresponds to a domain size of ~28 nm. The IT-4F binary blend film exhibits a scattering peak at *q* = 0.132 nm^−1^, corresponding to a domain size of ~24 nm. The position of the scattering peak moved to 0.124 nm^−1^ for the IT-DM/IT-4F ternary blend, which corresponds to a domain size of ~25 nm, indicating that the morphology of the IT-DM/IT-4F ternary blend originates from the interplay among the three components. The ITCF isomers blend shows a scattering peak at *q* = 0.183 nm^−1^, corresponding to a ~17 nm domain size, which is closer to the ideal exciton diffusion distance [60]. The IT-DM/IT-DM ternary blend showed scattering intensity higher than that of the IT-4F binary blend and comparable to that of the IT-DM binary blend. The ITCF isomers blend showed the highest scattering intensity among all the blends. The domain purity of the IT-DM/IT-4F blend is 0.708 relative to the ITCF blend. Thereby, higher domain purity and the relatively more ideal domain size of the ITCF isomers blend are responsible for lowered recombination and improved device performance [61].

Additionally, to evaluate the universality of the multicomponent solar cell based on small molecule acceptor isomers, we performed additional experiments. First, we studied the polymer effect by fabricating OSCs using ITCF isomers and other polymers. Two widely-used polymer donors (PBDB-T and PTB7-Th) rather than J71 were employed to fabricate two other multicomponent devices under the same conditions as the J71-based solar cells. We note that the PBDB-T and PTB7-Th (Scheme S2) are wide-bandgap and narrow-bandgap polymers, respectively. Similarly, control OSCs were also fabricated based on IT-DM/IT-4F and the donor PBDB-T or PTB7-Th. According to these experiments, ITCF isomers blend also demonstrated the highest FF and PCE compared with those of the reference IT-DM/IT-4F blend (Figures S6 and S7, Tables S5 and S6). We speculate that ITCF isomers may also demonstrate the more balanced charge transport as well as the favorable morphology in the active layer (blend with PBDB-T and PTB7-Th, respectively) compared to the reference IT-DM/IT-4F molecules, since the ITCF isomers-based devices demonstrated the highest FFs in devices compared to that of the reference IT-DM/IT-4F molecules in devices.

Second, we fabricated multicomponent solar cells based on different isomers (IXIC-2Cl isomers (Scheme S3), IT-M isomers (Scheme S4) and SY1 isomers [62] (Scheme S4)). As shown in Figure S8 and Table S7, the PBDB-T:IXIC-2Cl-based device exhibits a higher PCE (12.31%) than those of the devices based on IXIC (11.50%), IXIC-4Cl (11.15%) and IXIC:IXIC:4Cl (1:1, 11.58%). In addition, the multicomponent solar cell based on IT-M isomers and SY1 isomers have also same trend as the IXIC-2Cl isomers-based device (Figure S9 and Table S8). It is worth mentioning that the SY1-based device achieved a PCE as high as 18.35% with PM1 (Table S9) [63]. These results indicate that the improvement of device performance for the multicomponent solar cell based on acceptor isomers could be a general conclusion.

## 3. Materials and Methods

The details are shown in the Supplementary Materials. 

## 4. Conclusions

In summary, we emphasize the significance of using NFA isomers, which possess high structural homogeneity, to fabricate multicomponent solar cells through a qualitative comparison to the ternary solar cells based on non-isomeric NFAs with similar structural homogeneity. The ITCF isomers blend exhibited increased crystallinity and a more favorable domain size compared with the IT-DM/IT-4F ternary blend. The more favorable packing orientation (face-on) of the ITCF isomers is correlated with its more balanced charge transport, which, in conjunction with its increased exciton dissociation and decreased bimolecular recombination, contribute to its high FF and the consequent high PCE. These results demonstrate that using NFA isomers which possess high structural homogeneity to fabricate multicomponent solar cells could be an effective method to achieve a favorable morphology and thereby attain an improved device performance, which also could be a general methodology to boost the FF and PCE of non-fullerene organic solar cells.

## Figures and Tables

**Table 1:** Packing parameters for the pristine donor and acceptor films as well as the active blend films based on the GIWAXS measurements.

Samples	In Plane (100)			Out of Plane (010)		
	Location (Å^−1^)	d-Spacing (Å)	CL (nm)	Location (Å^−1^)	d-Spacing (Å)	CL (nm)
J71	0.2929	21.45	6.36	1.645	3.82	2.16
IT-DM	0.32237	19.49	10.05	1.7532	3.584	1.648
IT-4F	0.36462	17.23	3.41	1.4804	4.24	0.67
IT-DM:IT-4F	0.33091	18.988	15.409	1.7771	3.536	3.141
IT-CF	0.33185	18.93	14.36	1.7678	3.554	3.188
J71:IT-DM	0.3167	19.84	11.327	1.737	3.617	1.701
J71:IT-4F	0.31526	19.93	23.145	1.7412	3.609	2.182
J71:IT-DM:IT-4F	0.31706	19.82	14.267	1.7376	3.616	1.745
J71:IT-CF	0.309	20.29	5.245	1.753	3.58	2.164

## Supplementary Materials

The following supporting information can be downloaded at: https://www.mdpi.com/article/10.3390/molecules27185802/s1, Reference [50] is cited in the supplementary materials.

## Sample Availability

Samples of the compounds J71, IT-DM, IT-4F and ITCF are available from the authors.

## Abbreviations

ITCF	(2-(6-fluoro-5-methyl-3-oxo-2,3-dihydro-1*H*-inden-1-ylidene)malononitrile)-5,5,11,11-tetrakis(4-hexylphenyl)-dithieno[2,3-d:2′,3′-d′]-s-indaceno[1,2-b:5,6-b′]-dithiophene)
IT4F	3,9-bis(2-methylene-((3-(1,1-dicyanomethylene)-6,7-difluoro)-indanone))-5,5,11,11-tetrakis(4hexylphenyl)dithieno[2,3-d:2′,3′-d′]-s-indaceno[1,2-b:5,6-b′]dithiophene
J71	poly[4-(5-(4,8-bis(5-(tripropylsilyl)thiophen-2-yl)benzo[1,2-b:4,5-b′]dithiophen-2-yl)thiophen-2-yl)-5,6-difluoro-2-(2-hexyldecyl)-7-(thiophen-2-yl)-2*H*-benzo[d][1,2,3]triazole)]
