# Supplementary material for: Multicomponent Solar Cells with High Fill Factors and Efficiencies Based on Non-Fullerene Acceptor Isomers
Source: Molecules. 2022 Sep 7;27(18):5802. doi: 10.3390/molecules27185802 (PMC9504682; doi:10.3390/molecules27185802)
Supplement: Supplementary file 1 [file molecules-27-05802-s001.zip › molecules-1860934-supplementary.pdf]

molecules

MDPI

Article

Multicomponent Solar Cells with High Fill Factors and Efficiencies Based on Non-Fullerene Acceptor Isomers

Qiuning Wang [1,†], Yiwen Hou [2,†], Shasha Shi [3,4,†], Tao Yang [2,5,*], Ciyuan Huang [3,4], Shangfei Yao [3,4], Ziyang Zhang [3,4], Chenfu Zhao [3,4], Yudie Liu [3,4], Hui Huang [1], Lihong Wang [1], Chaoyue Zhao [1], Minghui Hao [6,*], Ye Tian [3,4,*], Bingsuo Zou [3,4,*] and Guangye Zhang [1,*]

[1] College of New Materials and New Energies, Shenzhen Technology University, Shenzhen 518118, China
[2] Julong College, Shenzhen Technology University, Shenzhen 518118, China
[3] Guangxi Key Laboratory of Processing for Nonferrous Metals and Featured Materials, Ministry of Education, School of Resources, Environments and Materials, Guangxi University, Nanning 530004, China
[4] Key Laboratory of New Processing Technology for Nonferrous Metals and Materials, Ministry of Education, School of Resources, Environments and Materials, Guangxi University, Nanning 530004, China
[5] Centre for Mechanical Technology and Automation, Department of Mechanical Engineering, University of Aveiro, 3810-193 Aveiro, Portugal
[6] Suzhou Key Laboratory of Advanced Lighting and Display Technologies, School of Electronic and Information Engineering, Changshu Institute of Technology, Changshu 215500, China
* Correspondence: yangtao@sztu.edu.cn (T.Y.); haomh2019@cslg.edu.cn (M.H.); tianye080t@163.com (Y.T.); zoubs@gxu.edu.cn (B.Z.); zhangguangye@sztu.edu.cn (G.Z.)
† These authors contributed equally to this work.

Solar Cell Fabrication and Characterization

Organic solar cells (OSCs) devices were fabricated with a structure of ITO/PEDOT:PSS/active layer/ZrAcAc/Al. ITO-coated glass substrates were cleaned by sonication in detergent, deionized water, acetone and isopropyl alcohol and dried under a nitrogen stream, followed by a UV-ozone treatment for 30 min. The J71:acceptors (weight ratio of 1:1) were dissolved in chloroform, then stirred overnight at room temperature to obtain a blend solution with a total concentration of 16 mg/mL. A thin PEDOT: PSS (Heraeus-Clevios P VP Al 4083) layer (40 nm) was spin-coated onto the ITO substrates and then dried at 150 °C for 15 min in air. The PEDOT:PSS coated ITO substrates were fast transferred to a N_2 filled glove-box for further processing. The blend solution was spin-cast on the top of PEDOT: PSS layer at 2000 rpm for 40 s. Then, it was annealed at 100 °C for 5 min. Subsequently, the active layer coated substrates were quickly transferred to a glove-box-integrated thermal evaporator for electrode deposition. A thin ZrAcAc layer and Al layer were sequentially evaporated under vacuum of 5×10^{-5} Pa through a shadow mask. The active area of each device was 5.9 mm^2 controlled by a shadow mask. The current-voltage (*J-V*) characteristic curves of all packaged devices were measured by using a Keithley 2400 Source Meter in air. Photocurrent was measured under AM 1.5G (100 mW cm^{-2}) using a Newport solar simulator in an Air. The light intensity was calibrated using a standard Si diode (with KG5 filter, purchased from PV Measurement) to bring spectral mismatch to unity. EQEs were measured using an Enlitech QE-S EQE system equipped with a standard Si diode. Monochromatic light was generated from a Newport 300W lamp source.

SCLC Measurements

The electron and hole mobility were measured by using the method of space-charge limited current (SCLC) for electron-only devices with the structure of ITO/ZnO/active layer/Zracac/Al and hole-only devices with the structure of ITO/MoOx/active layers/MoOx/Al. The charge carrier mobilities were determined by fitting the dark current to the model of a single carrier SCLC according to the equation: $J = 9\varepsilon_0\varepsilon_r\mu_h V^2/8L^3$, where J is

the current density, ε_0 is the permittivity of the free space, ε_r is the relative dielectric constant of the transport medium, μ_h is the hole mobility, V is the internal potential in the device, and L is the active layer thickness. The internal potential V is obtained by subtracting the built-in voltage (V_{bi}) and the voltage drop (V_s) from the series resistance of the substrate, from the applied voltage (V_{appl}), according to $V = V_{appl} - V_{bi} - V_s$.

Atomic Force Microscopy (AFM)

Atomic force microscopy (AFM) images were obtained by using a Dimension Icon AFM (Bruker) microscope in the tapping-mode.

GIWAXS Characterization

GIWAXS measurement were carried out with a Xeuss 2.0 SAXS/WAXS laboratory beamline using a Cu X-ray source (8.05 keV, 1.54 Å) and a Pilatus3R 300K detector. The incidence angle is 0.2°. The samples for GIWAXS measurements are fabricated on silicon substrates using the same recipe for the devices.

Resonant Soft X-ray Scattering

R-SoXS transmission measurements were performed at beamline 11.0.1.2 at the ALS. Samples for R-SoXS measurements were prepared on a PEDOT:PSS modified Si substrate under the same conditions used for OSC device fabrication, and then transferred by floating in water to a 1.5 mm × 1.5 mm, 100 nm thick Si3N4 membrane supported by a 5 mm × 5 mm, 200 mm thick Si frame (Norcada Inc.). Two-dimensional scattering patterns were collected on an in-vacuum CCD camera (Princeton Instrument PI-MTE). The beam size at the sample is 100 μm × 200 μm. The composition variation (or relative domain purity) over the length scales probed can be extracted by integrating scattering profiles to yield the total scattering intensity. The purer the average domains, the higher the total scattering intensity.

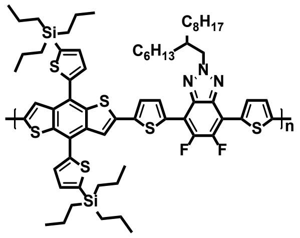

Scheme 1. Molecular structure of the donor polymer J71.

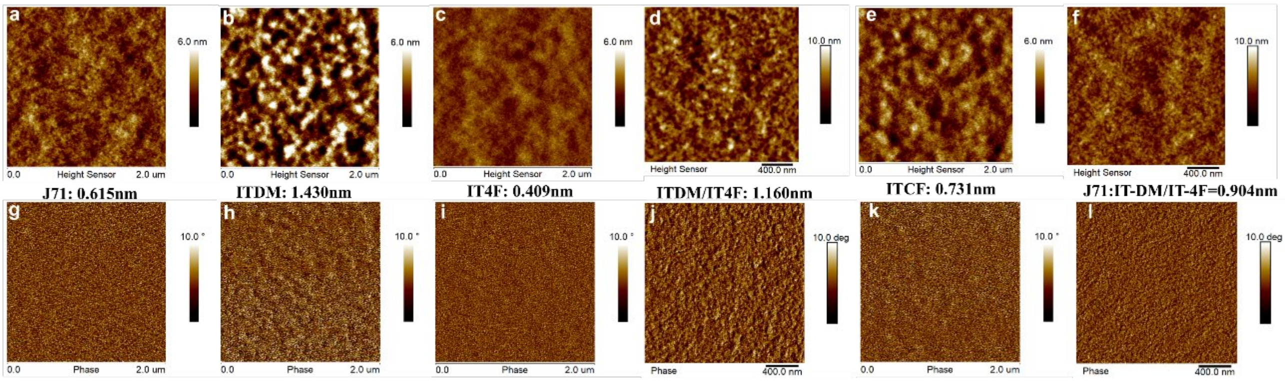

Figure S1. AFM Height (top) and Phase (bottom) Images of the Pristine Film for J71, IT-DM, IT-4F, IT-DM/IT-4F, ITCF, and J71: IT-DM/IT-4F. J71, IT-DM, IT-4F, IT-DM/IT-4F, ITCF adapted with permission from ref [1]. Copyright (2019) American Chemical Society. These data are used here just to make the comparison more visible for the ease of readers.

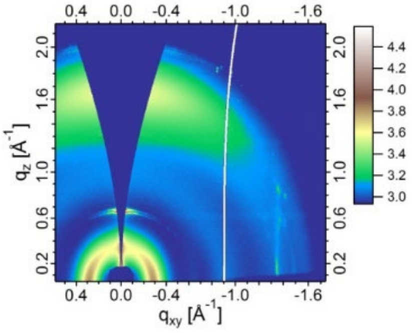

Figure S2. The GIWAXS 2D patterns of the pristine films of J71.

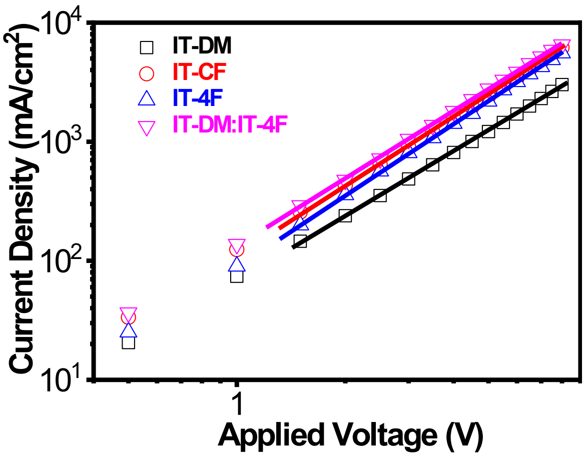

Figure S3. Electron SCLC mobilities of the acceptor films.

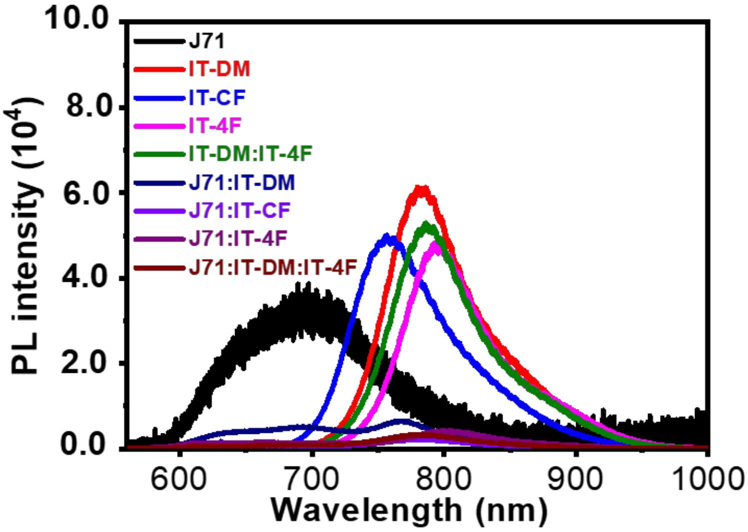

Figure S4. Photoluminescence spectra of the pristine films and the active blend films (excited at 633 nm).

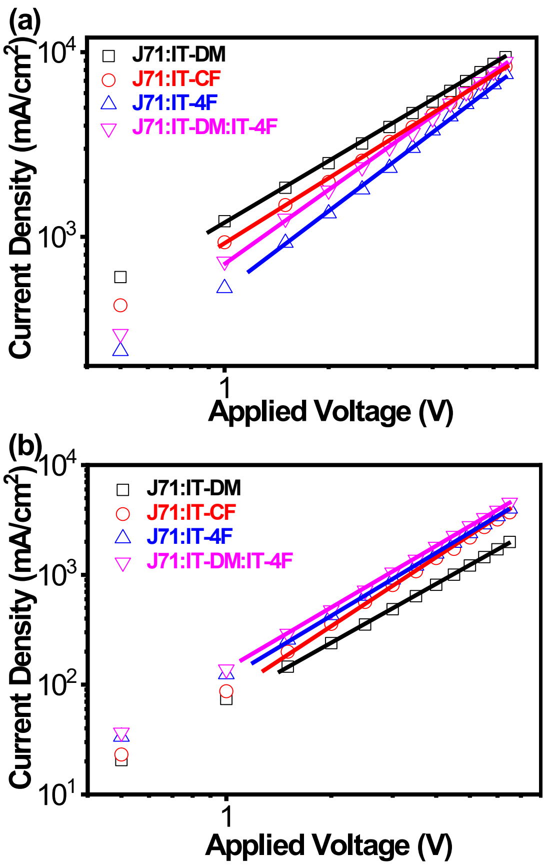

Figure S5. Hole (**a**) and electron (**b**) SCLC mobilities of the active blends.

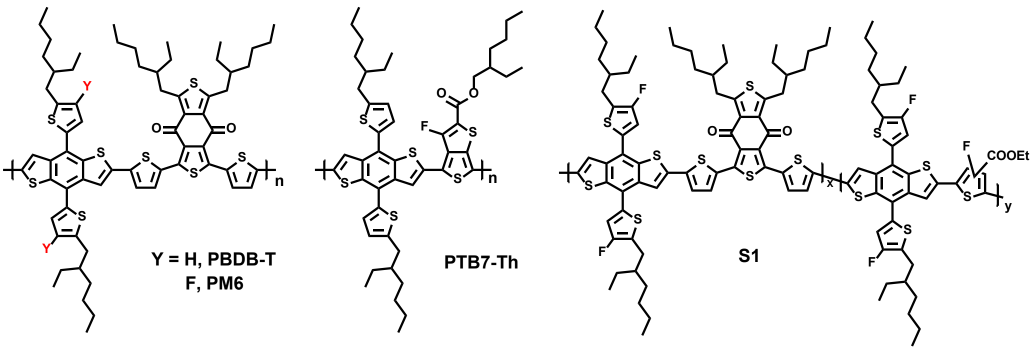

Scheme 2. Molecular structure of PBDB-T, PTB7-Th, S1 and PM6.

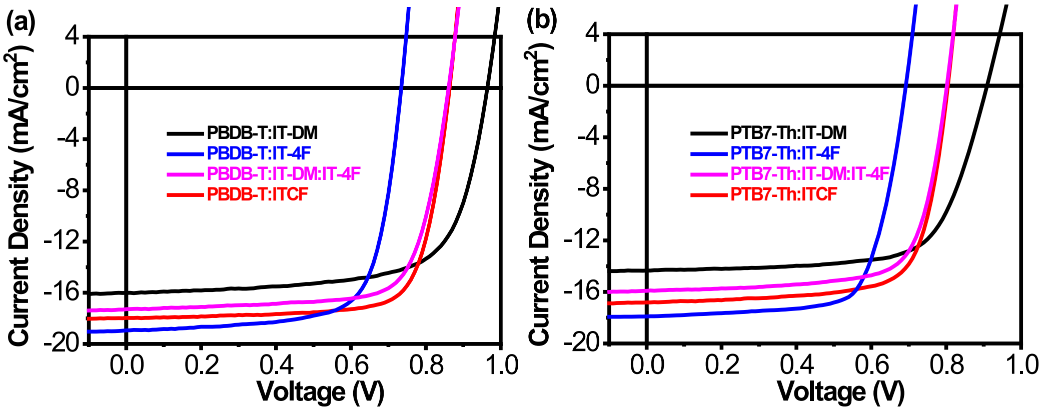

Figure S6. *J-V* curves of the OSC devices based on the blend of (**a**) acceptor/PBDB-T; (**b**) acceptor/PTB7-Th.

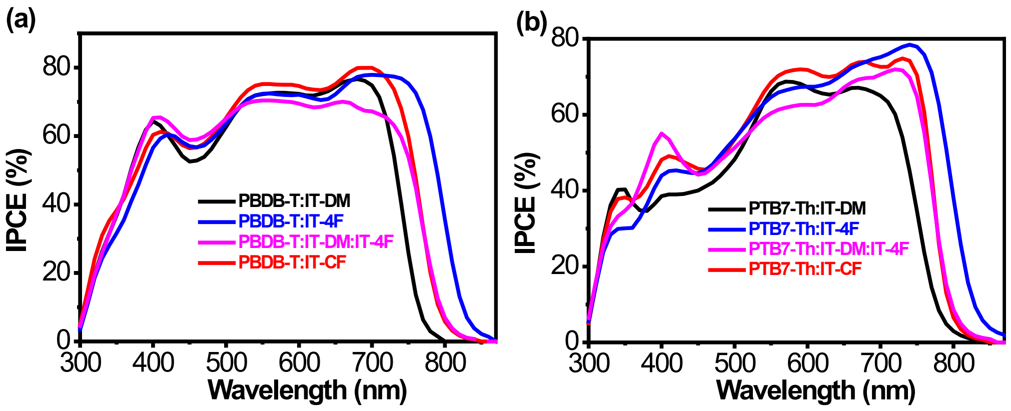

Figure S7. IPCE spectra of the OSC devices based on the blend of (**a**) acceptor/PBDB-T; (**b**) acceptor/PTB7-Th.

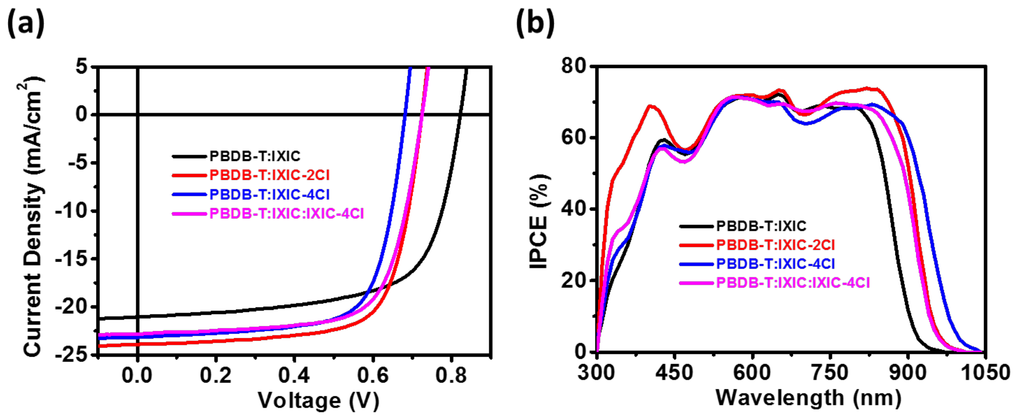

Figure S8. (**a**) *J-V* Curves of the Optimized Devices; (**b**) IPCE Spectra of the Optimized Devices.

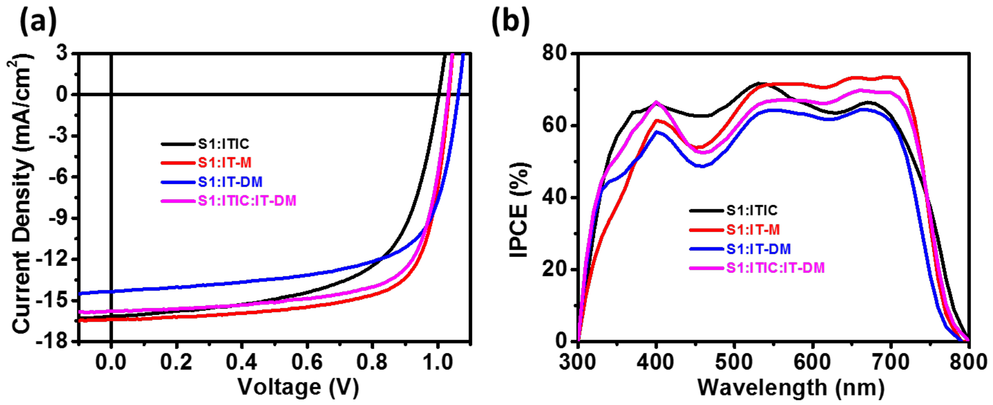

Figure S9. (**a**) *J-V* Curves of the Optimized Devices; (**b**) IPCE Spectra of the Optimized Devices.

Table S1. The SCLC mobility parameters of the different films.

Samples	μ_h (cm^2 V^{-1} s^{-1})	μ_e (cm^2 V^{-1} s^{-1})	μ_h/μ_e
**J71**	0.0011031	/	/
**IT-DM**	/	0.0006876	/
**IT-4F**	/	0.0007928	/
**IT-DM:IT-4F**	/	0.0007289	/
**ITCF**	/	0.0007461	/
**J71:IT-DM**	0.0008002	0.0004026	1.9876
**J71:IT-4F**	0.0008806	0.0005026	1.7521
**J71:IT-DM/IT-4F**	0.0008680	0.0004918	1.7649
**J71:ITCF**	0.0008307	0.0005298	1.5680

Table S2. Optimal photovoltaic parameters of solar cells under standard AM 1.5G illumination, 100 mW cm^{-2}.

Blends	V_{OC} (V)	J_{SC} (mA/cm^2)	FF	PCE (%)	PCE$_{max}$ (%)
**J71:IT-DM**	1.010 ± 0.005	16.30 ± 0.23	0.702 ± 0.007	11.56 ± 0.24	11.74
**J71:IT-CF**	0.902 ± 0.004	18.49 ± 0.29	0.764 ± 0.005	12.74 ± 0.26	13.35
**J71:IT-4F**	0.800 ± 0.006	19.48 ± 0.28	0.738 ± 0.006	11.50 ± 0.23	11.60
**J71:IT-DM:IT-4F**	0.900 ± 0.005	17.52 ± 0.26	0.735 ± 0.006	11.59 ± 0.24	11.92

Table S3. Photovoltaic parameters calculated from the J_{ph}-V_{eff} curves of the studied devices.

Blends	J_{sat}^{a} (mA cm^{-2})	J_{ph}^{b} (mA cm^{-2})	J_{ph}^{c} (mA cm^{-2})	J_{ph}^{b}/J_{sat} (%)	J_{ph}^{c}/J_{sat} (%)
**J71:IT-DM**	17.573	16.378	13.589	93.2	77.3
**J71:IT-4F**	20.804	19.544	17.014	93.9	81.8
**J71:IT-DM:IT-4F**	18.861	17.877	15.402	94.7	81.7
**J71:IT-CF**	19.786	18.915	17.209	95.6	86.9

[a] J_{sat} is the J_{ph} under condition of V_{eff} = 2.0 V; [b] The J_{ph} under short-circuit condition; [c] The J_{ph} under maximum power output condition.

Table S4. Summary of the active blend morphology characteristics measured with R-SoXS.

Blends	Domain purity	Location (nm^{-1})	Domain Size (nm)
**J71:IT-DM**	0.702	0.110	28.418
**J71:IT-4F**	0.563	0.132	23.816
**J71:IT-DM/IT-4F**	0.708	0.124	25.213
**J71:IT-CF**	1	0.183	17.199

Table S5. Photovoltaic parameters of PBDB-T based OSCs under AM 1.5G illumination, 100 mW cm^{-2}.

Samples	V_{OC}(V)	J_{SC} (mA/cm^2)	FF	PCE (%)
**PBDB-T:IT-DM**	0.964	16.01/15.90[a]	0.693	10.700
**PBDB-T:IT-CF**	0.863	17.96/17.68[a]	0.758	11.756
**PBDB-T:IT-DM:IT-4F**	0.861	17.29/16.64[a]	0.737	10.966
**PBDB-T:IT-4F**	0.734	18.94/18.80[a]	0.719	10.002

[a] J_{SC} integrated from the IPCE curves.

Table S6. Photovoltaic parameters of PTB7-Th-based OSCs under AM 1.5G illumination, 100 mW cm^{-2}.

Samples	V_{OC}(V)	J_{SC} (mA/cm²)	FF	PCE (%)
**PTB7-Th:IT-DM**	0.909	14.34/14.04[a]	0.694	9.041
**PTB7-Th:IT-4F**	0.692	17.90/17.64[a]	0.717	8.884
**PTB7-Th:IT-DM:IT-4F**	0.802	15.91/15.52[a]	0.725	9.255
**PTB7-Th:IT-CF**	0.804	16.81/16.47[a]	0.729	9.860

[a] J_{SC} integrated from the IPCE curves.

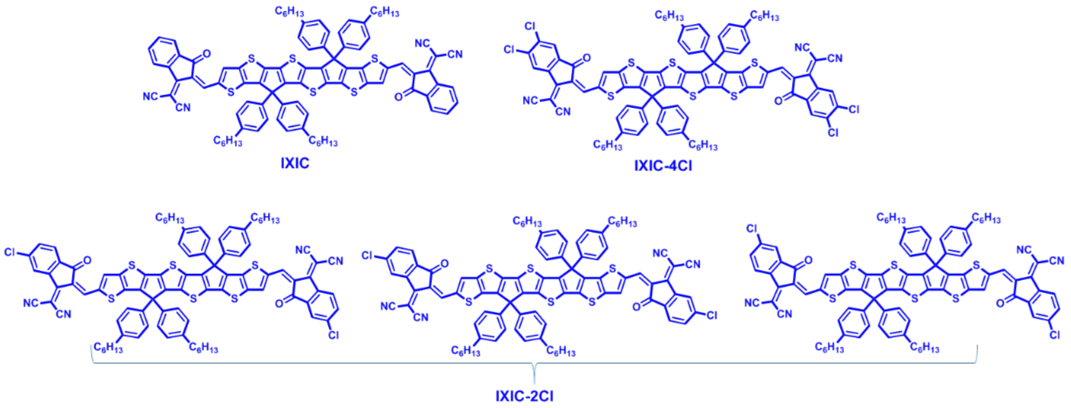

Scheme 3. Molecular structure of IXIC, IXIC-4Cl and IXIC-2Cl isomers.

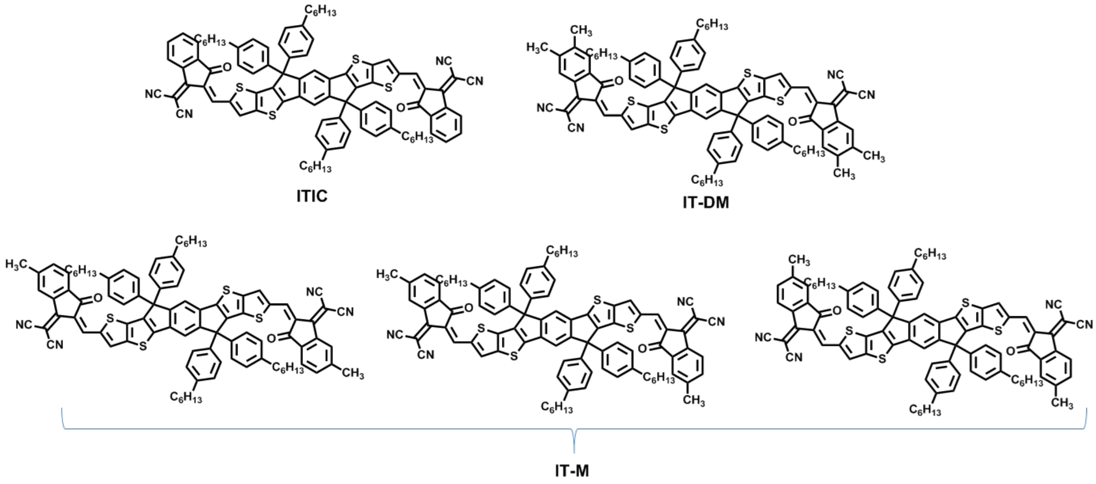

Scheme 4. Molecular structure of ITIC, IT-DM and IT-M isomers.

Table S7. Photovoltaic parameters of PBDB-T-based OSCs under AM 1.5G illumination, 100 mW cm^{-2}.

Samples	V_{OC}(V)	J_{SC} (mA/cm²)	FF	PCE (%)
**PBDB-T:IXIC**	0.822	21.04/20.92[a]	0.665	11.499
**PBDB-T:IXIC-4Cl**	0.681	23.17/23.03[a]	0.706	11.146
**PBDB-T:IXIC:IXIC-4Cl**	0.724	22.83/22.28[a]	0.701	11.583
**PBDB-T:IXIC-2Cl**	0.724	23.90/23.66[a]	0.711	12.310

[a] J_{SC} integrated from the IPCE curves.

Table S8. Photovoltaic parameters of S1-based OSCs under AM 1.5G illumination, 100 mW cm^{-2}.

Samples	V_{OC}(V)	J_{SC} (mA/cm^2)	FF	PCE (%)
**S1:ITIC**	1.002	16.18/15.70[a]	0.616	9.987
**S1:IT-DM**	1.062	14.35/13.80[a]	0.660	10.060
**S1:ITIC:IT-DM**	1.031	15.80/15.41[a]	0.709	11.551
**S1:IT-M**	1.033	16.41/15.97[a]	0.714	12.113

[a]J_{SC} integrated from the IPCE curves.

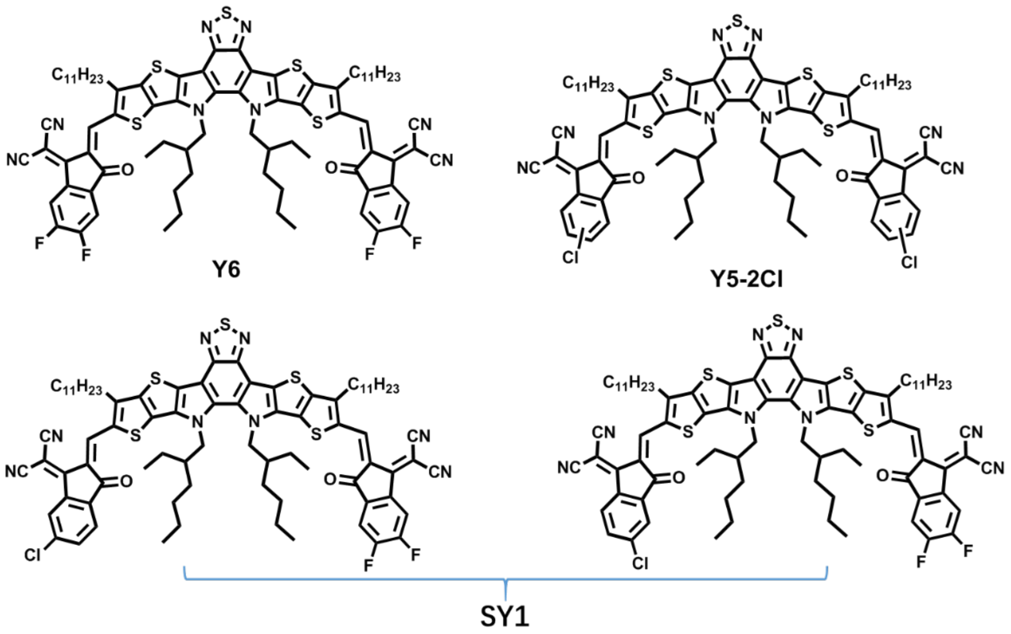

Scheme 5. Molecular structure of Y6, Y5-2Cl and SY1 isomers.

Table S9. Photovoltaic parameters of PM1-based OSCs under AM 1.5G illumination, 100 mW cm^{-2}.

Samples	V_{OC}(V)	J_{SC} (mA/cm^2)	FF	PCE (%)
**PM1:Y6**	0.861	26.35/26.03[a]	0.771	17.493
**PM1: Y5-2Cl**	0.917	24.35/23.86[a]	0.708	15.808
**PM1:SY1:Y5-2Cl**	0.888	26.09/25.66[a]	0.743	17.216
**PM1:SY1**	0.889	26.33/25.90[a]	0.784	18.349

[a]J_{SC} integrated from the IPCE curves.

References

1. Hao, M.; Liu, T.; Xiao, Y.; Ma, L.-K.; Zhang, G.; Zhong, C.; Chen, Z.; Luo, Z.; Lu, X.; Yan, H.; et al. Achieving Balanced Charge Transport and Favorable Blend Morphology in Non-Fullerene Solar Cells via Acceptor End Group Modification. *Chem. Mater.* **2019**, *31*, 1752–1760. https://doi.org/10.1021/acs.chemmater.8b05327.
